# Curcumin Alleviates Endometriosis in Sexually Mature Female Mice by Targeting PKA and Inhibiting De Novo Estrogen Synthesis

**DOI:** 10.3390/biomedicines14092099

**Published:** 2026-09-17

**Authors:** Haiyang Zhao, Xudong Chen, Sirui Zhang, Jieyi Zhang, Yueyuan Wang, Zhengrong Xia, Hui Wang, Zhaohui Xu

**Affiliations:** 1 Innovative Chinese Medicine Research Institute, Engineering Research Center of Modern Preparation Technology of Traditional Chinese Medicine, Ministry of Education, Shanghai University of Traditional Chinese Medicine, Shanghai 201203, China; 2 State Key Laboratory of Reproductive Medicine and Offspring Health, Department of Histology and Embryology, School of Basic Medical Sciences, Nanjing Medical University, Nanjing 211166, China; 3 National Demonstration Center for Experimental Basic Medical Education, Nanjing Medical University, Nanjing 211166, China; 4 Analysis and Testing Center, Nanjing Medical University, Nanjing 211166, China

**Keywords:** curcumin, endometriosis, protein kinase A, de novo estrogen synthesis, drug-target binding, BALB/c mice

## Abstract

**Background:** Endometriosis is an estrogen-dependent disorder with limited treatment options. The precise molecular targets of curcumin and its effects on local estrogen synthesis in this disease remain unclear. **Methods:** We investigated curcumin’s efficacy and mechanism via network pharmacology, in vitro functional assays in ectopic endometrial cells, an allogeneic mouse model, and target validation through molecular docking, thermal shift, and hydrolysis stability assays. **Results:** Curcumin significantly inhibited ectopic cell malignant phenotypes, reducing migration distance by 55.60 ± 7.32%, 80.34 ± 11.19%, and 82.88 ± 13.67% at 5, 10, and 15 μM, respectively, and decreasing invasive cell numbers by 40.38 ± 7.67%, 58.33 ± 12.70%, and 60.18 ± 9.07%, respectively (all *p* < 0.05). In a mouse model, curcumin (200 mg/kg/day) reduced lesion number by 38.46% (2.167 ± 0.752 vs. 1.333 ± 0.516, *p* = 0.0493) and lesion volume by 59.73% (0.0497 ± 0.0173 vs. 0.0200 ± 0.0130 mm^3^, *p* = 0.00732). It also suppressed de novo estrogen synthesis and modulated the estrogen receptor α (ERα) and β (ERβ) ratio. Mechanistically, curcumin directly bound to and inhibited protein kinase A (PKA), a key kinase in the cAMP signaling pathway. **Conclusions:** We first demonstrate that curcumin alleviates endometriosis by targeting PKA to inhibit local estrogen synthesis, identifying PKA as a novel therapeutic target and curcumin as a promising treatment candidate.

## 1. Introduction

Endometriosis (EMs) is an estrogen-dependent gynecological disorder characterized by the presence of endometrial glands and stroma outside the uterine cavity. Globally, EMs affects approximately 10% of women of reproductive age, and it is diagnosed in 30–50% of women with infertility [1,2]. The disease manifests clinically as progressive dysmenorrhea, chronic pelvic pain, and dyspareunia and significantly impairs quality of life [3]. More critically, studies have identified a significantly elevated risk of premature mortality among endometriosis patients, underscoring its long-term threat to women’s health [4]. Furthermore, endometriosis is strongly associated with infertility; approximately 50% of affected women face fertility challenges, and postoperative recurrence rates remain high, ranging from 20% to 50%, imposing substantial psychological and economic burdens on patients and their families [5]. Current medical therapies for EMs primarily include progestins (e.g., dienogest), aromatase inhibitors, and GnRH agonists, and their long-term use is limited by adverse effects such as osteoporosis, hot flushes, and metabolic disturbances. Further, recurrence is common after drug withdrawal [6]. Thus, there is an urgent need for safer and more effective therapeutic strategies.

The pathogenesis of endometriosis is complex and involves multifactorial interactions. The classic theory of “retrograde menstruation” is considered foundational; however, only about 10% of women with retrograde menstruation develop endometriosis, suggesting that local microenvironmental and immune dysregulation play crucial roles in disease initiation and progression [7,8]. Recent studies have elucidated the core pathological features of endometriosis, including the aberrant migratory and invasive capacities of ectopic endometrial cells which, coupled with hyperproliferative and anti-apoptotic properties, enable them to breach the basement membrane barrier, implant at distant sites, and sustain growth [9]. Critically, endometriosis is definitively established as an estrogen-dependent condition. Estrogen, binding to its receptors (ERα and ERβ), drives proliferation, angiogenesis, and neuroinvasion within ectopic lesions [10]. The recently proposed “estrogen microenvironment” theory further posits that locally elevated “de novo synthesis” of estrogen within ectopic tissues is a key driver. Key enzymes such as aromatase (CYP19A1) and 17β-hydroxysteroid dehydrogenase (HSD17B) are highly expressed in lesions, leading to elevated estrogen levels and creating autocrine/paracrine loops that exacerbate disease progression [11]. Additionally, an imbalance in estrogen receptor subtype ratios, particularly the relative overexpression of ERβ, can alter tissue sensitivity to estrogen, promoting inflammation and fibrosis [12]. Emerging evidence also implicates reproductive tract microbiota and miRNAs in EMs pathogenesis [13,14].

Given the limitations of existing therapies, natural products and traditional medicines offer promising alternatives due to their multi-targeting potential and favorable safety profiles. Traditional Chinese Medicine (TCM) possesses a long history of managing endometriosis. The disease is categorized under “dysmenorrhea” and “abdominal mass” in TCM, with its core pathogenesis attributed to “blood stasis and obstruction.” The recorded functions of *Curcuma longa* L. in the Compendium of Materia Medica (“Ben Cao Gang Mu”)—namely, “breaking blood, moving qi, unblocking channels, and relieving pain”—align closely with this pathogenesis, suggesting its therapeutic potential for endometriosis [15]. Modern pharmacological research demonstrates that curcumin, the primary active constituent of turmeric, exhibits potent anti-inflammatory, antioxidant, and anti-proliferative properties, potentially mediated through modulating pathways like NF-κB and MAPK [16]. Previous studies confirm that curcumin can inhibit the proliferation and invasion of endometriotic cells, with mechanisms linked to the regulation of the PI3K/AKT/GSK-3β signaling pathway [17]. Clinical reports indicate that TCM formulations containing turmeric improve EMs-associated pain [18,19,20]. Furthermore, evidence suggests curcumin can ameliorate endometriosis symptoms by regulating miRNA expression, offering novel insights for elucidating its molecular mechanisms [21]. However, given the pivotal role of estrogen in EMs pathogenesis and progression, whether curcumin can modulate estrogen levels and associated responses to inhibit endometriosis development remains an open question warranting investigation.

Based on this rationale, we integrated network pharmacology and bioinformatics to predict the association between curcumin and EMs. We then used in vitro cellular assays and an allogeneic mouse model to validate curcumin’s therapeutic efficacy. Finally, we investigated whether curcumin suppresses de novo estrogen synthesis and identified its direct molecular target. This study aims to elucidate the mechanism of curcumin’s anti-EMs action and provide experimental evidence for developing targeted natural-product-based therapies.

## 2. Materials and Methods

### 2.1. Network Pharmacological Analysis

#### 2.1.1. Identifying Curcumin Targets

The SMILES notation of curcumin (O=C(C=CC1=CC=C(O)C(OC)=C1)CC(=O)C=CC2=CC=C(O)C(OC)=C2) was retrieved from the PubChem database (https://pubchem.ncbi.nlm.nih.gov/) (accessed on 2 April 2025), and potential protein targets of curcumin were predicted using the SwissTargetPrediction database (http://swisstargetprediction.ch/ accessed on 2 April 2025).

#### 2.1.2. Identifying Endometriosis Targets

Disease targets associated with endometriosis (EMs) were retrieved from the GeneCards database (https://www.genecards.org/) (accessed on 15 November 2025), and targets with a Relevance score greater than the median value were selected for further analysis. The R package “VennDiagram” was utilized to identify the overlapping targets between curcumin and EMs, and a Venn diagram was generated to visualize the intersection.

#### 2.1.3. Constructing Protein–Protein Interaction (PPI) Network and Screening of Key Targets

The identified overlapping targets were uploaded to the STRING database (https://string-db.org/) (accessed on 16 November 2025) for PPI network analysis. The organism was set to “Homo sapiens,” with a minimum required interaction score of 0.4 (moderate confidence). Disconnected nodes were hidden in the network. The resulting PPI network was subsequently imported into Cytoscape (version 3.9.1) software for further visualization and refinement. Using the cytoHubba plugin, the degree values of each node were calculated based on the Degree algorithm, and the top 10 key targets (hub genes) were filtered out in descending order of degree values.

#### 2.1.4. Gene Ontology (GO) and Kyoto Encyclopedia of Genes and Genomes (KEGG) Pathway Enrichment Analysis

GO and KEGG pathway enrichment analyses were performed on the overlapping targets using the R (version 4.3.1) packages “enrichplot” and “ggplot2”. GO enrichment analysis encompassed three categories: Biological Process (BP), Cellular Component (CC), and Molecular Function (MF). The enriched terms were sorted by *p*-value, and the top 10 significantly enriched terms from each category were selected for visualization.

#### 2.1.5. Molecular Docking

The three-dimensional (3D) crystal structures of the target proteins were obtained in PDB format from the RCSB Protein Data Bank (https://www.rcsb.org/) (accessed on 13 April 2025). Using PyMOL (version 2.5) software, water molecules were removed from the target protein structures, and the original ligands were extracted. Hydrogen atoms were added to the proteins using AutoDock Tools, and the files were saved in PDBQT format. The 3D structure of the active drug component (curcumin) was retrieved from the PubChem database in SDF format and subsequently converted to PDBQT format using PyMOL and AutoDock Tools. Finally, molecular docking between the target proteins and the ligand was performed using AutoDock Vina (version 1.2.3) to determine the optimal binding conformation and binding energy.

#### 2.1.6. Gene Set Enrichment Analysis (GSEA)

To investigate the relationship between hormone-related pathways and EMs, Gene Set Enrichment Analysis (GSEA) was performed using the R package “clusterProfiler”. The gene expression dataset for EMs (GSE58178) was downloaded from the GEO database. Gene sets related to hormone pathways were obtained from the MSigDB database (specifically, the C2: KEGG_LEGACY subset of CP and C5: GO Gene Ontology gene sets).

### 2.2. In Vitro Experiments

#### 2.2.1. Reagents and Cell Culture

Curcumin (purity ≥ 98%, determined by high-performance liquid chromatography, HPLC) was purchased from Sigma-Aldrich (Cat. No. C1386, St. Louis, MO, USA). It was dissolved in dimethyl sulfoxide (DMSO) for in vitro experiments, with the final concentration of DMSO in the culture medium maintained below 0.1% (*v*/*v*) to avoid cytotoxicity. For in vivo animal experiments, curcumin was suspended in 0.5% sodium carboxymethyl cellulose (CMC-Na) solution for oral gavage administration.

The human endometriotic stromal cell line (12Z) and complete culture medium were purchased from Nanjing Yifeixue Biotechnology Co., Ltd. (Nanjing, China). Cells were cultured at 37 °C in a humidified atmosphere containing 5% CO_2_, and those in good growth condition were seeded into culture dishes and allowed to adhere overnight. Subsequently, the cells were treated with curcumin at concentrations of 5, 10, and 15 μM for specified time points.

#### 2.2.2. Cell Viability Assay

Cell Counting Kit-8 (CCK-8) was purchased from Beyotime Biotechnology (C0038, Shanghai, China). Cells were seeded into 96-well plates at a density of 5000 cells per well, and after the respective treatments, 10 μL of CCK-8 solution was added to each well. Following incubation at 37 °C for 2 h, the absorbance was measured at a wavelength of 450 nm using a microplate reader (Bio-Rad, Hercules, CA, USA).

#### 2.2.3. Proliferation/Apoptosis Detection

An Annexin V-FITC Apoptosis Detection Kit (C1062M) was purchased from Beyotime Biotechnology. Cells were seeded into 6-well plates and subjected to different treatments, after which they were collected, washed with pre-cooled PBS, and centrifuged. The cell pellet was resuspended in 195 μL of 1× Annexin V Binding Buffer to prepare a single-cell suspension, which was transferred to flow cytometry tubes. Then, 5 μL of FITC-Annexin V and 10 μL of Propidium Iodide (PI) staining solution were added, mixed gently, and incubated on ice in the dark for 10–15 min. Samples were then analyzed using a flow cytometer.

#### 2.2.4. Migration Assay

Target cells in the logarithmic growth phase were harvested, and the cell concentration was adjusted. They were uniformly seeded into 6-well plates and cultured in complete medium until they reached 80–90% confluence, forming a confluent monolayer. Using a sterile 200 μL pipette tip, two straight, parallel scratches of uniform width were created in each well. The detached cells were gently washed away with PBS twice, and the medium was replaced with serum-free medium. The plates were then returned to the incubator for continued culture. The scratch areas were photographed under a microscope at 0, 24, and 48-h time points, and the scratch width was measured using ImageJ software (Version 1.54p).

#### 2.2.5. Invasion Assay

Transwell chambers were coated with Matrigel (354234, Corning Incorporated, Corning, NY, USA) and incubated for 30 min. Cells in the logarithmic phase were trypsinized and resuspended in serum-free medium, and 200 μL of the cell suspension (containing 5000 cells) was added to the upper chamber. The lower chamber was filled with 600 μL of complete medium containing serum. After 48 h of incubation, the chambers were removed, washed twice with PBS, fixed with 4% paraformaldehyde for 20 min, and stained with 0.1% crystal violet (C0121) for 30 min. Cells that had invaded through the Matrigel to the lower surface of the membrane were counted in five randomly selected fields under a microscope.

#### 2.2.6. Immunofluorescence (IF)

Paraffin-embedded tissue sections were baked at 65 °C for 2 h. Deparaffinization and rehydration were performed sequentially using xylene I, xylene II, absolute ethanol I, absolute ethanol II, 90% ethanol, 80% ethanol, and 70% ethanol. Antigen retrieval was performed, and sections were fixed with pre-cooled immunofluorescence fixative for 20 min. After washing three times, sections were permeabilized with 0.1% Triton X-100 (Biotechnology, Shanghai, China) for 5 min and blocked with 1% Bovine Serum Albumin (BSA) at room temperature for 1 h. Sections were then incubated with primary antibodies at 4 °C overnight, followed by incubation with fluorescently labeled secondary antibodies at room temperature for 1 h. Cell nuclei were counterstained with DAPI for 1 h.

#### 2.2.7. Western Blotting (WB)

After treatment, the cells were lysed in RIPA buffer containing protease inhibitors, and the protein concentration was determined via a BCA assay (Beyotime, Shanghai, China). Equal amounts of protein (30 µg) were separated by 10% SDS–PAGE and transferred to nitrocellulose membranes. The membranes were blocked with 5% skim milk for 1 h and incubated overnight at 4 °C with the following primary antibodies: ER-β (1:1000), VEGFA (1:3000), CYP19a1 (1:1000), StAR (1:1000), LHCGR (1:1000), PKA (1:1000), cAMP (1:500), and GAPDH (1:50,000) (all from Proteintech, Wuhan, China). HRP-conjugated secondary antibodies (1:1000; Beyotime, Shanghai, China) were applied using 5% non-fat milk for 1 h at room temperature. The protein bands were visualized via enhanced chemiluminescence reagents (FuDeBio, Hangzhou, China) and analyzed via Quantity One software (Bio-Rad, Hercules, CA, USA) (Version 4.6.7).

#### 2.2.8. Thermal Stability Assay

Protein lysates from different treatment groups were centrifuged. The supernatant was collected and aliquoted equally into six PCR tubes, with the tubes labeled and heated at 37, 47, 57, 67, 77, and 87 °C for 5 min, after which they were centrifuged. The supernatant from each tube was transferred to new PCR tubes, ensuring equal volume. Loading buffer was added, and the samples were boiled for 5 min, followed by Western blot analysis. A thermal denaturation curve was plotted based on the remaining protein levels.

#### 2.2.9. Hydrolysis Stability Assay

Cells were lysed to extract total protein. After centrifugation, the supernatant was collected and aliquoted equally into six PCR tubes, which were labeled, and proteinase K was added to the tubes along with different concentrations of the test drug (curcumin). The control group received no additions, while the positive control group received proteinase K only. The tubes were incubated on a shaker for 30 min. Loading buffer was then added, and the samples were boiled for 5 min, followed by Western blot analysis to assess protein stability.

### 2.3. Animal Experiments

#### 2.3.1. Animal Housing

Specific pathogen-free (SPF) 8-week-old sexually mature female BALB/c mice were purchased from Beijing Vital River Laboratory Animal Technology Co., Ltd. (Beijing, China) [Laboratory Animal Production License No.: SCXK (Zhe) 2019-0004]. All mice were acclimatized for one week prior to experimentation. The study protocol was approved by the Animal Ethics Committee of Nanjing Medical University (IACUC-2402036), and all experimental procedures were strictly conducted in accordance with the relevant animal welfare guidelines and regulations.

#### 2.3.2. Dose Selection and Rationale

The oral dose of curcumin (200 mg/kg/day) was selected based on previously published preclinical studies investigating the therapeutic effects of curcumin in gynecological disease models, including EMs. Previous studies have demonstrated that this dose exhibits significant therapeutic efficacy against EMs in mouse models, with no observed systemic toxicity or adverse effects on body weight, organ index, or reproductive function. In addition, pharmacokinetic studies have shown that this dose can achieve effective drug concentrations in the peritoneal cavity and uterine tissues of mice, which is critical for targeting ectopic endometriotic lesions [22] (Li et al., 2025). The dose was also converted to the human equivalent dose (HED) based on body surface area, which corresponds to a clinically acceptable oral dose of curcumin in humans (16 mg/kg/day), consistent with the dose range used in previous clinical trials of curcumin for chronic inflammatory diseases [12].

#### 2.3.3. Animal Modeling and Drug Administration

Mice were randomly divided into three groups (n = 6 per group): the Model group, the Curcumin group, and the Sham-operated group. Mice in the Curcumin group received daily oral gavage of curcumin (200 mg/kg/day) for one week at a fixed time each day, with body weight monitored and, on day 8, an EMs model was established using a tissue fragment intraperitoneal injection method. The donor-to-recipient mouse ratio was 1:2 and the donor mice were euthanized by cervical dislocation. Under aseptic conditions, the abdominal cavity was opened to harvest adipose tissue and uterine horns. The Y-shaped uterus was opened longitudinally to expose the endometrium, which was then cut at the bifurcations, minced into approximately 1 mm^3^ fragments using ophthalmic scissors, and suspended in 0.6 mL of pre-warmed (37 °C) sterile saline for injection. Recipient mice were anesthetized, and the abdominal skin was disinfected. A suspension containing the uterine tissue fragments was injected into the peritoneal cavity of the recipient mice using a 1 mL syringe fitted with a 16-gauge needle, entering at a point 0.5 cm above the urethral opening in the lower midline abdomen. The injection site was swabbed with iodine to prevent infection. Mice in the Sham-operated group received an intraperitoneal injection of an equal volume of adipose tissue fragments from donor mice, following the same procedure. After modeling, administration of curcumin to the Curcumin group continued for one additional week. On the day following the final gavage, all mice were euthanized by cervical dislocation for sample collection.

Mice weighed 18–22 g at the start of the experiment; they were housed in an SPF facility at 22 ± 2 °C and 50 ± 10% humidity, with a 12 h light/dark cycle and 4–5 mice per cage, and free access to food and water. Randomization was performed using a random number table; all surgeries were conducted under deep anesthesia; and animals were euthanized by cervical dislocation at the experimental endpoint to minimize suffering.

#### 2.3.4. Ectopic Lesion Assessment

The location, morphology, color, and size of endometriotic lesions in the mouse abdominal cavity were observed and recorded. Lesions were carefully excised, and their length, width, and height were measured using a caliper. Lesion volume was calculated using the formula: Volume = Length × Width × Height. The lesions were then fixed in 4% paraformaldehyde for 24 h at room temperature, followed by routine paraffin embedding, sectioning, and hematoxylin and eosin (H&E) staining to observe the pathological morphology.

### 2.4. Statistical Analysis

The data are presented as means ± standard deviations (SDs). All normalized data passed the normality test and statistical analyses were conducted via GraphPad Prism 10. Differences between groups were evaluated by Student’s *t*-test or one-way ANOVA, with *p* < 0.05 considered statistically significant.

## 3. Results

**Figure 1:**
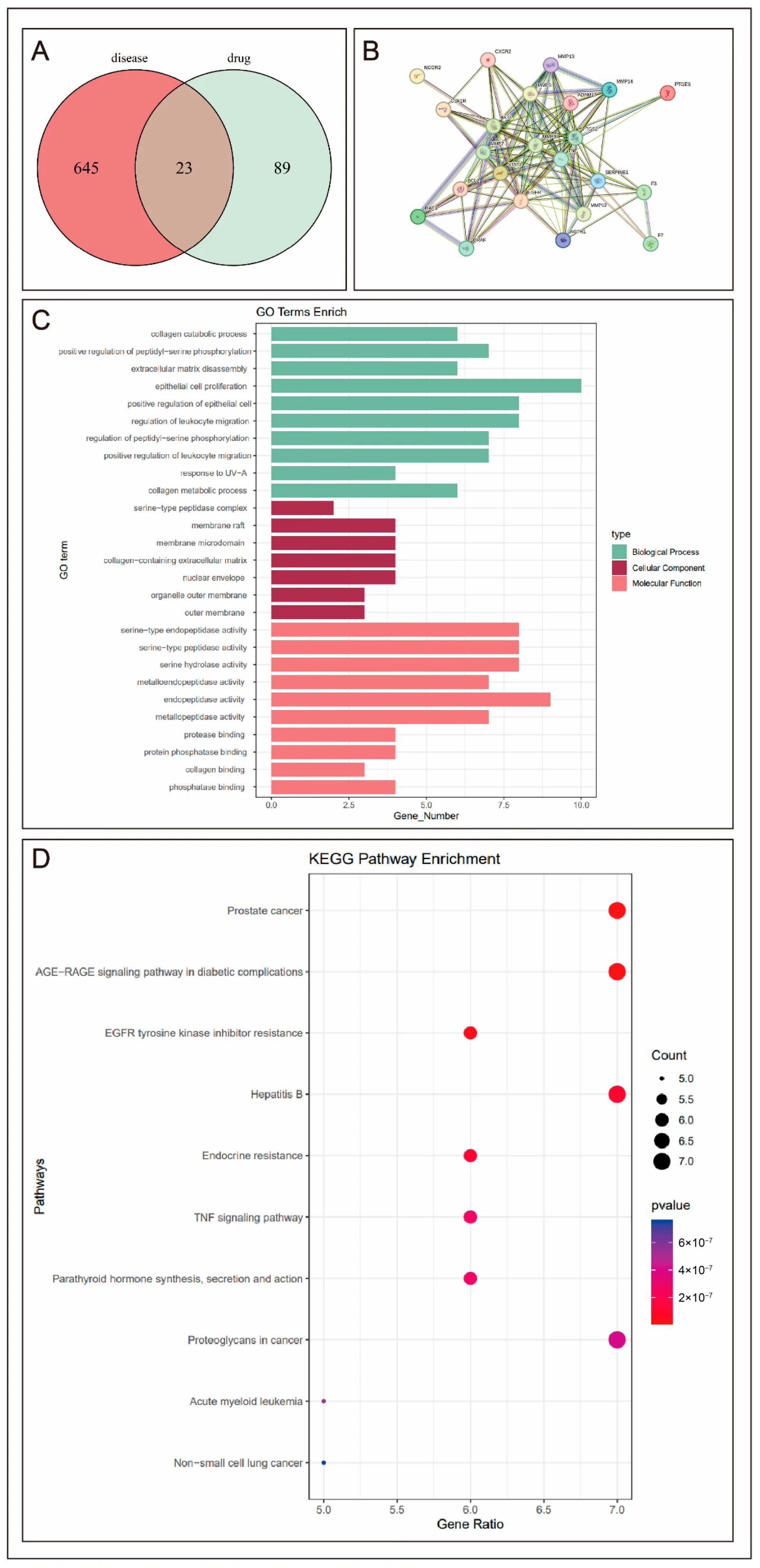
Network pharmacological analysis of the association between curcumin and EMs. (**A**) Venn diagram of the overlapping targets between curcumin and endometriosis. (**B**) Protein–protein interaction (PPI) network of the common targets. (**C**) Gene Ontology (GO) enrichment analysis. (**D**) Kyoto Encyclopedia of Genes and Genomes (KEGG) pathway enrichment analysis. Subsequently, the inhibitory effect of curcumin on ectopic endometrial cells was evaluated in vitro using the ectopic endometrial cell line (12Z) and a healthy endometrial epithelial cell line (HEE). The cytotoxic effects of curcumin on 12Z and HEE cells were assessed via the CCK-8 cell viability assay. Results indicated that curcumin, within the concentration range of 5–20 μM, did not significantly inhibit the proliferation of 12Z cells on Day 1 (*p* > 0.05). Continued treatment still did not exhibit significant toxicity on Day 2, with cell viability remaining above 85% at concentrations below 20 μM (Figure 2A). Based on these findings, the concentration range of 5–15 μM was determined to be suitable for subsequent curcumin treatment in 12Z cells.

Initially, data mining and network pharmacological analysis were conducted to evaluate the potential therapeutic value of curcumin (Cur) in endometriosis (EMs). Based on SwissTargetPrediction, 112 potential targets of curcumin were identified, which were intersected with 668 EMs-related targets retrieved from the Genecards database, resulting in 23 common targets. Using a protein–protein interaction (PPI) network, the top 10 key targets were identified (MMP9, TNF, PTGS2, AKT1, EGFR, STAT3, SERPINE1, MMP3, ADAM17, and MMP7) (Figure 1A,B). Functional enrichment analysis revealed that these key targets were primarily associated with crucial pathological processes in EMs, such as the inflammatory response, extracellular matrix remodeling, and estrogen signaling regulation (Figure 1C,D). This suggests that curcumin may exert its effects by synergistically targeting multiple pathways to inhibit the invasion and proliferation of ectopic endometrial cells, as well as the formation of a local estrogenic microenvironment.

**Figure 2:**
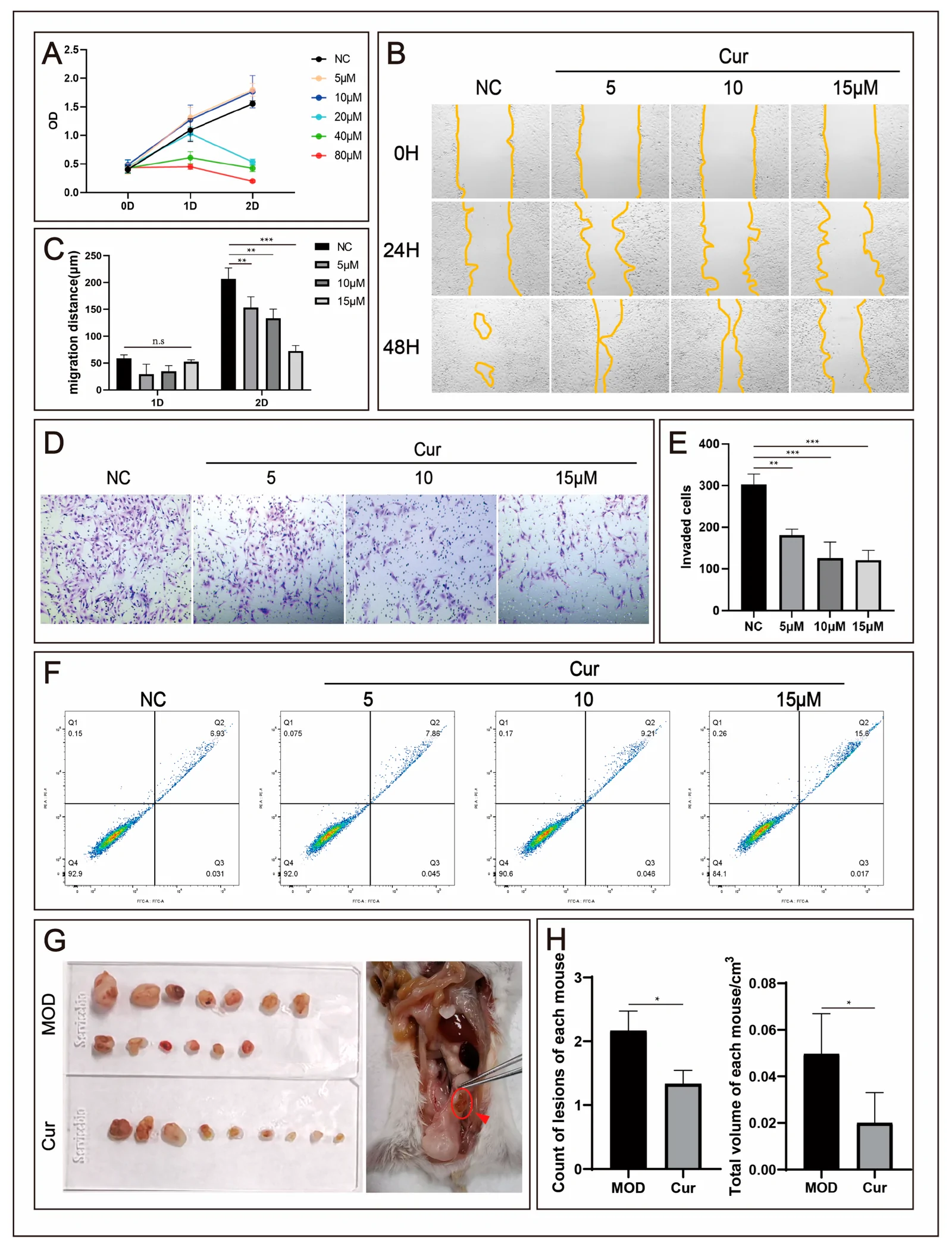
In vitro and in vivo therapeutic effects of curcumin intervention on endometriosis. (**A**) Determination of appropriate curcumin intervention concentrations. (**B**) Analysis of cell invasion capability. (**C**) Quantification of the wound-healing assay. (**D**) Analysis of cell migration capability, scale = 10 μm. (**E**) Quantification of Transwell invasion assay. (**F**) Detection of apoptosis levels by flow cytometry. (**G**) Representative photographs of ectopic endometrial lesions. Red arrow and circle: EMs lesions. (**H**) Statistical analysis of ectopic lesions. (n = 3 vitro experiments; n = 6 animal experiments; *: *p* < 0.05, **: *p* < 0.01, ***: *p* < 0.001, ns: *p* > 0.05).

Wound healing and Transwell invasion assays demonstrated that curcumin significantly and dose-dependently inhibited the migratory and invasive capabilities of 12Z cells. Compared to the control group, treatment with 5, 10, and 15 μM curcumin for 24 h reduced the migration distance of 12Z cells by 55.60 ± 7.32%, 80.34 ± 11.19%, and 82.88 ± 13.67%, respectively (*p* < 0.05). Similarly, the number of invading cells was reduced by 40.38 ± 7.67%, 58.33 ± 12.70%, and 60.18 ± 9.07%, respectively (*p* < 0.05) (Figure 2B–E).

Consistently, flow cytometric analysis revealed that curcumin also promoted apoptosis in 12Z cells in a significant dose-dependent manner (Figure 2F). Furthermore, in vivo efficacy was validated in a mouse allogeneic transplantation EMs model. After one week of intervention with curcumin (200 mg/kg/day), the curcumin-treated group showed a significant reduction in the number of ectopic lesions by 38.46% (2.167 ± 0.752 vs. 1.333 ± 0.516, *p* = 0.0493) and a decrease in lesion volume by 59.73% (0.0497 ± 0.0173 vs. 0.0200 ± 0.0130 mm^3^, *p* = 0.00732) compared to the control group (n = 6) (Figure 2G,H). Histomorphological examination of the excised lesions confirmed that the tissues exhibited typical features of ectopic endometrium, as shown in Supplementary Figure S1.

**Figure 3:**
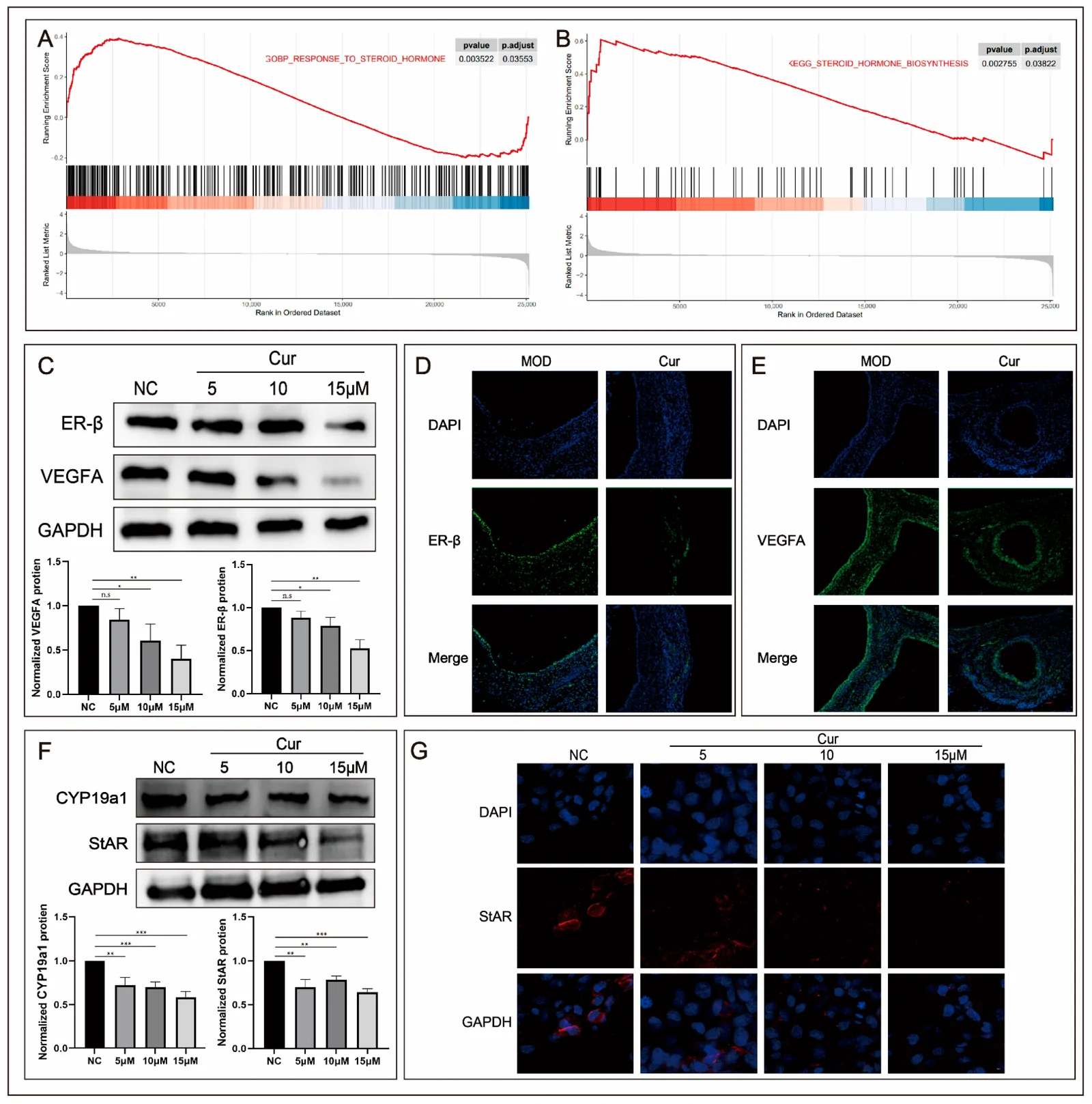
GSEA and biological event analysis of curcumin intervention in EMs. GSEA plots for (**A**) steroid hormone response and (**B**) steroid hormone biosynthesis in EMs. (**C**) Western blot analysis of estrogen sensitivity and angiogenesis-related proteins. Immunofluorescence staining of (**D**) estrogen receptors and (**E**) vascular endothelial growth factor. (**F**) Western blot analysis of estrogen synthesis-related proteins. (**G**) Immunofluorescence staining of estrogen synthesis-related proteins. (n = 3; *: *p* < 0.05, **: *p* < 0.01, ***: *p* < 0.001, ns: *p* > 0.05). (**D**,**E**): scale bar = 50 μm; (**G**): scale bar = 5 μm.

Building upon the confirmed therapeutic efficacy, Gene Set Enrichment Analysis (GSEA) revealed significant enrichment of hormone-related gene sets (RESPONSE_TO_STEROID_HORMONE and STEROID_HORMONE_BIOSYNTHESIS) in EMs samples, suggesting a high relevance of steroid hormone synthesis and signaling to EMs development (Figure 3A,B). Combined with existing literature and GSEA results, it was established that systemic estrogen levels and cellular estrogen sensitivity are key biological events involved in regulating EMs pathogenesis.

Western blot (WB) and immunofluorescence (IF) analyses demonstrated that compared to normal endometrial tissues, the protein levels of ER-β and the vascular growth factor VEGFA were significantly elevated in both ectopic endometrial cells and lesions. Curcumin treatment markedly suppressed this increase, indicating that curcumin effectively counteracts the aberrantly elevated estrogen sensitivity and angiogenesis characteristic of EMs (Figure 3C–E).

Furthermore, the expression of key proteins involved in estrogen synthesis was examined. Results showed that curcumin significantly inhibited the upregulation of aromatase (CYP19A1) and 17β-hydroxysteroid dehydrogenase type 1 (HSD17B1) in ectopic endometrial cells (Figure 3F,G). This demonstrates that curcumin effectively suppresses the local estrogen synthesis capacity within EMs tissues.

**Figure 4:**
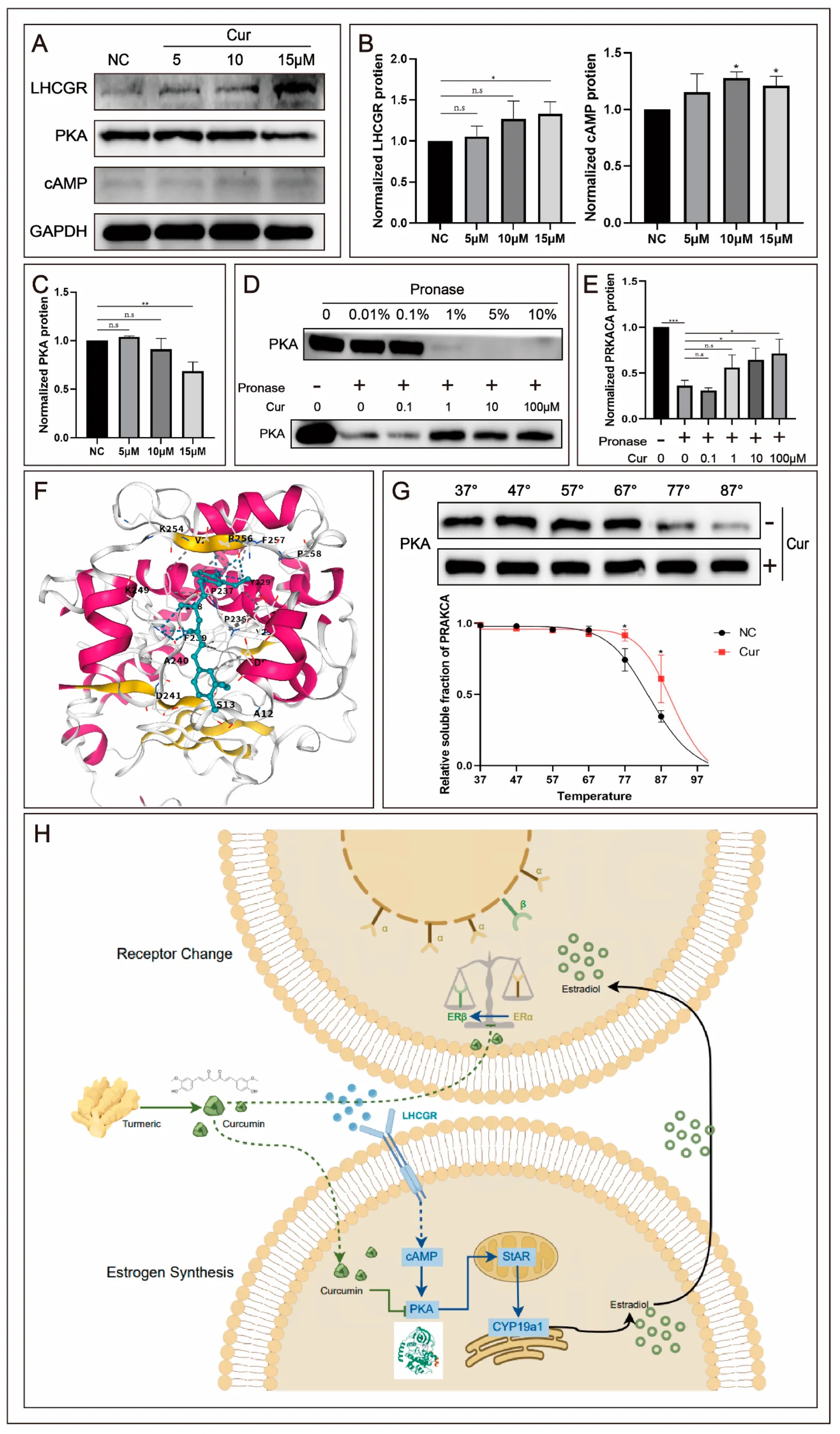
Curcumin-targeted regulation of estrogen synthesis and receptor switching. (**A**) Western blot analysis of proteins in the estrogen synthesis pathway. Densitometric quantification of (**B**) LHCGR/cAMP pathway proteins and (**C**) PKA protein levels from Western blots. (**D**) Optimization of hydrolysis enzyme concentration and analysis of hydrolysis stability. (**E**) Statistical analysis of the hydrolysis stability of the curcumin–PKA complex. (**F**) Molecular docking analysis of curcumin with PKA. (**G**) Thermal stability analysis of the curcumin–PKA complex. (**H**) Schematic diagram illustrating the proposed mechanism of curcumin targeting. (n = 3; *: *p* < 0.05, **: *p* < 0.01, ***: *p* < 0.001, ns: *p* > 0.05).

Subsequently, the regulatory effect of curcumin on estrogen synthesis and its binding target was further validated through molecular docking, thermal shift, and hydrolysis stability assays. Western blot analysis indicated that curcumin significantly suppressed the protein expression of PKA within the LHCGR-cAMP pathway (Figure 4A–C). Molecular docking analysis demonstrated that curcumin stably binds within the ATP-binding pocket of the PKA catalytic subunit, with a predicted binding energy of −6.9 kcal/mol, which is comparable to that of the known PKA inhibitor H89 (−7.2 kcal/mol). This suggests a strong binding affinity of curcumin for PKA (Figure 4F).

Both the hydrolysis stability and thermal stability assay results confirmed a high binding interaction between curcumin and PKA (Figure 4D,E,G).

In summary, curcumin targets PKA to regulate estrogen synthesis, while concurrently and specifically inhibiting the shift in estrogen receptor subtype predominance (from α to β). This dual action ultimately leads to reduced estrogen secretion capacity and diminished estrogen sensitivity in ectopically implanted endometrial cells (Figure 4H).

## 4. Discussion

While previous studies using various models have indicated the potential therapeutic value of curcumin in EMs **,** existing research has predominantly focused on its anti-inflammatory effects mediated through pathways like NF-κB and MAPK, often lacking elucidation of precise molecular mechanisms [17]. Although recent reports suggest curcumin inhibits EMs cell proliferation via the PI3K/AKT/GSK-3β pathway, a targeted investigation into its effects on estrogen signaling—a pivotal aspect of EMs pathogenesis—has remained absent.

This study is the first to systematically elucidate the molecular mechanism by which curcumin alleviates endometriosis by targeting PKA and inhibiting de novo estrogen synthesis. Recent advances have further expanded our understanding of endometriosis pathogenesis and the clinical application of curcumin, providing a broader context for our findings. Lamceva et al. comprehensively reviewed the main pathogenetic theories, including retrograde menstruation, immune dysregulation, stem cell involvement, and epigenetic alterations, highlighting the multifactorial nature of the disease [21]. Carbone et al. emphasized the critical roles of molecular signaling and programmed cell death pathways in endometriosis, suggesting that these pathways represent promising avenues for future curative treatments [23]. Griffiths et al. summarized recent advances that could accelerate diagnosis and improve patient care, underscoring changes in hormonal, inflammatory, and pain pathways as key contributors to disease etiology [24]. Furthermore, single-cell RNA sequencing studies have provided fresh insights into the pathogenesis of endometriosis at the cellular level, identifying epithelial cell clusters as potential progenitors of ectopic lesions and revealing immune cell deficiencies in clearing ectopic endometrial cells [25].

Our investigational strategy was initiated with a network pharmacology approach, which unbiasedly predicted the close association between curcumin and EMs, guiding our subsequent mechanistic exploration. We not only confirmed the therapeutic efficacy of curcumin against EMs but also robustly validated its specific binding to PKA through multiple orthogonal approaches, including hydrolysis stability assays, thermal shift assays, and molecular docking. This work establishes a complete “curcumin–PKA–estrogen synthesis–EMs” axis of action, identifying a novel potential therapeutic target for clinical EMs management. Integration of network pharmacology, in vitro cellular experiments, and in vivo animal model data demonstrated that curcumin significantly inhibits the migration, invasion, and proliferation of ectopic endometrial cells and markedly reduces lesion number and volume in a mouse allogeneic transplantation model, which was selected to minimize individual variability and enhance reproducibility for drug efficacy evaluation [26]. The dosage of curcumin (200 mg/kg/day) was chosen based on previous pharmacokinetic and efficacy studies establishing its tolerability and effectiveness in murine models, and our in vitro dose–response results confirmed the biological relevance of the concentrations used [22,27]. Mechanistically, curcumin specifically targets PKA and, via modulation of the PKA-cAMP signaling pathway, suppresses the synthesis and expression of aromatase (CYP19A1) and the steroidogenic acute regulatory protein (StAR). This leads to reduced local estrogen levels and an altered ERα/ERβ ratio, thereby decreasing the estrogen sensitivity of endometrial cells and effectively disrupting key processes in EMs initiation and progression. These findings not only reveal a specific mechanism of action for curcumin but also provide new insights for developing targeted EMs therapies based on natural products.

From the perspective of modernizing Traditional Chinese Medicine (TCM), this study offers novel insights. TCM theory categorizes EMs under “abdominal mass” and “dysmenorrhea”, with its pathogenesis attributed to “blood stasis and obstruction,” warranting a therapeutic principle of “promoting blood circulation and resolving stasis”. *Curcuma longa* L. is documented in the Compendium of Materia Medica (Ben Cao Gang Mu) for its abilities to promote “blood circulation, regulate qi flow, unblock meridians, and relieve pain.” While modern foundational research has gradually uncovered the potential therapeutic value of curcuminoids for female diseases, including EMs, analysis of modern clinical medication patterns does not frequently list *Curcuma longa* L. in traditional EMs formulations (Supplementary Table S1), suggesting a potential disconnect between certain traditional empirical knowledge and modern scientific discovery. Building on this, our study employed a data-driven approach to precisely identify the active component curcumin from *Curcuma longa* L., predicted its molecular mechanism using network pharmacology, and subsequently validated its efficacy and specific mechanism through cellular and animal models. This strategy provides a replicable pathway for investigating other reproductive system diseases and exploring the application of TCM herbs where classical theoretical support might be limited.

From a translational medicine standpoint, this study holds significant clinical value. While current EMs drugs like dienogest are effective, long-term use can lead to adverse effects including osteoporosis and hot flashes [18]. By contrast, curcumin, as a natural product, demonstrates a favorable safety and tolerability profile. Preclinical studies have shown no significant toxicity in mice at oral doses of 1500 mg/kg/day, and clinical trials for various conditions have confirmed its safety in humans [16]. Future work could explore curcumin as an adjuvant or alternative therapy in EMs management, particularly for patients desiring fertility or those intolerant to existing medications. Furthermore, the identification of PKA as a target provides a new direction for developing novel EMs therapeutics.

However, this study has several limitations. First, while network pharmacology screened curcumin as the potential active component, comprehensive phytochemical studies are lacking to systematically document the compound screening process from the crude herb. While Traditional Chinese Medicine research typically selects herbs based on clinical experience, our medication pattern analysis for EMs did not find a significant use of *Curcuma longa* L. This divergence from conventional practice indicates that its potential efficacy, uncovered by modern research, may need to be reconciled with or expand upon existing TCM theory. Second, the low oral bioavailability of curcumin was not addressed, potentially hindering clinical translation. Third, discrepancies exist between animal models and human EMs; the allogeneic transplantation model used here, while simulating some disease features, cannot fully replicate human disease complexity. Fourth, the study primarily focused on the PKA-cAMP pathway, whereas curcumin likely acts through multiple targets; future studies should explore its comprehensive network of actions.

Addressing these limitations, future research should focus on (1) developing curcumin nano-delivery systems to enhance its bioavailability and targeting; (2) utilizing patient-derived xenograft (PDX) models or organoids to establish more clinically relevant EMs models; (3) applying proteomics and metabolomics to comprehensively map curcumin’s effects; (4) conducting prospective clinical trials to evaluate curcumin’s safety and efficacy in EMs patients.

Notably, several of the hub genes identified in our network pharmacology analysis have been previously implicated in endometriosis by genetic association studies. Large-scale genome-wide association studies (GWASs) have established TNF and EGFR as endometriosis-associated genes, while MMP9 has been repeatedly linked to endometriosis susceptibility in candidate gene studies. Although genes such as STAT3, MMP3, ADAM17, MMP7, and SERPINE1 have not been directly identified as susceptibility loci in GWAS, their protein products are well-recognized for their functional roles in inflammation, extracellular matrix remodeling, and angiogenesis—processes central to endometriosis pathogenesis. Furthermore, the estrogen biosynthesis genes CYP19A1 and HSD17B1, which we found to be suppressed by curcumin, have been consistently associated with endometriosis in both candidate gene studies and GWASs. This convergence between our network pharmacology-predicted targets and independently established genetic evidence strengthens the biological relevance of our findings and supports the notion that curcumin may act through pathways with established genetic underpinnings in endometriosis.

In conclusion, this study elucidates the molecular mechanism by which curcumin alleviates EMs via targeting PKA and inhibiting de novo estrogen synthesis, providing a new paradigm for modernizing traditional medicine research. Despite its limitations, this work opens new avenues for EMs-targeted therapy and highlights the significant potential of TCM-derived approaches. With advancing research, precision therapies based on active components of Chinese herbs could become important strategies in EMs management, potentially benefiting millions of patients worldwide.

## Data Availability

The data that support the findings of this study are available from the corresponding author upon reasonable request.

## Supplementary Materials

The following supporting information can be downloaded at: https://www.mdpi.com/article/10.3390/biomedicines14092099/s1, Figure S1: Histopathological examination by haematoxylin and eosin (H&E) staining confirmed successful establishment of the endometriosis model in the model group (MOD), where ectopic lesions displayed typical endometrial morphology with well-defined glandular structures lined by columnar epithelial cells and abundant surrounding stroma, as observed at both ×4 and ×40 magnifications. In the curcumin-treated group (Cur), there appeared to be a tendency towards reduced glandular density, mild glandular atrophy, and decreased stromal components compared to the MOD group; however, these morphological differences did not reach statistical significance, likely due to the limited sample size and the qualitative nature of the histological assessment; Table S1: Screening of active components in Curcumaelongae Rhizoma.

## Abbreviations

Abbreviation	Full Name
EMs	Endometriosis
Cur	Curcumin
PKA	Protein Kinase A
cAMP	Cyclic Adenosine Monophosphate
ERα	Estrogen Receptor Alpha
ERβ	Estrogen Receptor Beta
CYP19A1	Aromatase
HSD17B1	17β-Hydroxysteroid Dehydrogenase Type 1
StAR	Steroidogenic Acute Regulatory Protein
LHCGR	Luteinizing Hormone/Choriogonadotropin Receptor
VEGFA	Vascular Endothelial Growth Factor A
TCM	Traditional Chinese Medicine
GO	Gene Ontology
KEGG	Kyoto Encyclopedia of Genes and Genomes
PPI	Protein–Protein Interaction
GSEA	Gene Set Enrichment Analysis
H&E	Hematoxylin and Eosin
WB	Western Blotting
IF	Immunofluorescence
CCK-8	Cell Counting Kit-8
DMSO	Dimethyl Sulfoxide
HPLC	High-Performance Liquid Chromatography
SD	Standard Deviation
SPF	Specific Pathogen-Free
