# Supplementary material for: Curcumin Alleviates Endometriosis in Sexually Mature Female Mice by Targeting PKA and Inhibiting De Novo Estrogen Synthesis
Source: Biomedicines. 2026 Sep 17;14(9):2099. doi: 10.3390/biomedicines14092099 (PMC13604060; doi:10.3390/biomedicines14092099)
Supplement: Supplementary file 1 [file biomedicines-14-02099-s001.zip › Supplementary Figure.pdf]

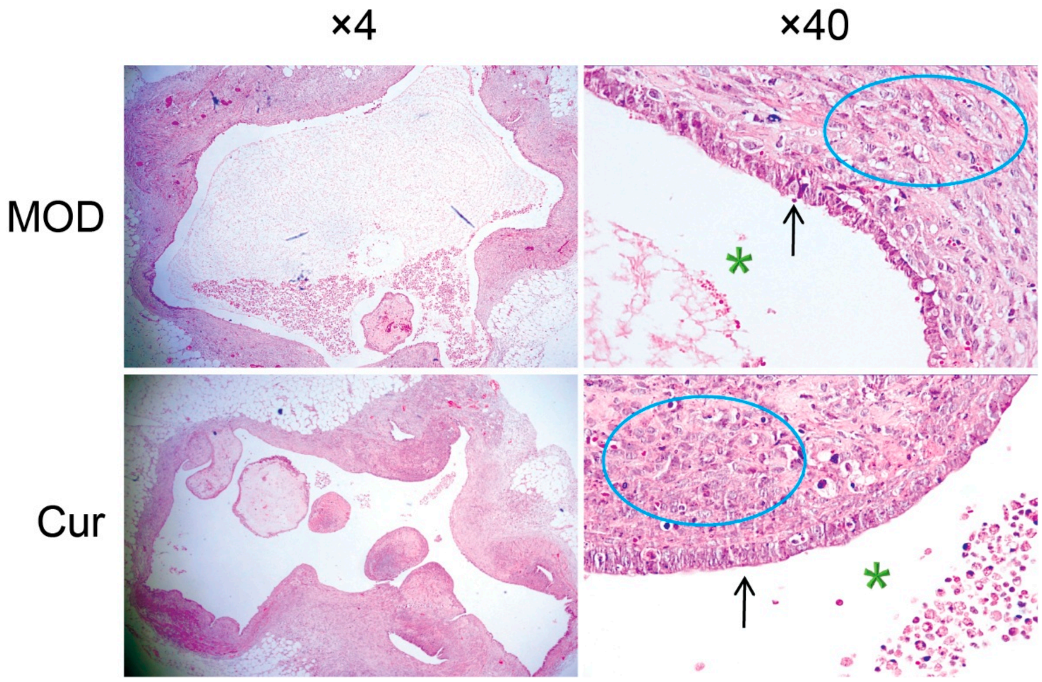


* (green): cystic cavity; Arrow (black): Columnar epithelial cells; Circle (blue): stromal cells

Figure S1: Histopathological examination by haematoxylin and eosin (H&E) staining confirmed successful establishment of the endometriosis model in the model group (MOD), where ectopic lesions displayed typical endometrial morphology with well-defined glandular structures lined by columnar epithelial cells and abundant surrounding stroma, as observed at both ×4 and ×40 magnifications. In the curcumin-treated group (Cur), there appeared to be a tendency towards reduced glandular density, mild glandular atrophy, and decreased stromal components compared to the MOD group; however, these morphological differences did not reach statistical significance, likely due to the limited sample size and the qualitative nature of the histological assessment.
